# Increased expression of TAZ and associated upregulation of PD-L1 in cervical cancer

**DOI:** 10.1186/s12935-021-02287-y

**Published:** 2021-11-04

**Authors:** Yanyan Han, Dandan Liu, Lianhong Li

**Affiliations:** 1 Department of Pathology, Okayama University Graduate School of Medicine, Dentistry, and Pharmaceutical Sciences, 2-5-1, Shikata-cho, Kita-ku, Okayama, 700-8558 Japan; 2 The Fourth Medical Center of The General Hospital of the Chinese People’s Liberation Army, Beijing, 100048 China; 3 Pathology Department of Dalian Medical University, Liaoning, 116044 China

**Keywords:** TAZ, PD-L1, Cervical cancer

## Abstract

**Background:**

As an important component of the Hippo pathway, WW domain-containing transcription regulator 1 (TAZ), is a transcriptional coactivator that is responsible for the progression of various types of cancers. Programmed cell death protein 1 (PD-1) receptors in activated T cells and their ligand programming death force 1 (PD-L1) are the main checkpoint signals that control T cell activity. Studies have shown high levels of PD-L1 in various cancers and that PD-L1/PD-1 signals to evade T-cell immunity. Recent data have demonstrated that TAZ can regulate the characteristics of cancer cells via PD-L1. Cervical cancer is a common gynecological disease worldwide. In this study, we attempted to evaluate the effects of TAZ and PD-L1 on cervical cancer.

**Methods:**

Hela cervical cancer cells were transfected with TAZ plasmid or TAZ siRNA or PD-L1 siRNA by using Lipofectamine 2000. The relationship between TAZ and PD-L1 in cervical cancer cells was determined by qRT-PCR and western blotting. The functional roles of TAZ were confirmed via CCK-8, Transwell and flow cytometry assays. Western blotting was utilized to observe the expression of BCL-2 and Caspase-3. The clinicopathological correlation of TAZ and PD-L1 was evaluated via relevant databases.

**Result:**

TAZ is upregulated in cervical cancer and induces the growth and metastasis of cervical cancer cells by targeting PD-L1and inhibiting the ratio of apoptotic of cancer cells. High TAZ and PD-L1 expression was observed in different stage, grade, histological patterns, and ages of cervical cancer groups compared with normal cervix groups. Furthermore, high TAZ expression was positively correlated with the infiltration levels of immune cells and the expression of PD-L1.

## Introduction

Cervical cancer (CC) is the second most common cancer among women in the worldwide and it has a high mortality rate [1]. Although significant achievements have been made in CC therapy, its prognosis of it is still poor due to the high metastasis and recurrence [2]. Thus, it is necessary to find new treatment methods and molecular mechanisms for CC.

As an evolutionary preservation pathway, the Hippo signaling pathway can control the size of organs and regulate cell proliferation, self-renewal, differentiation, and survival [3]. Moreover, Hippo signaling can have two important roles, tumor-suppressive and oncogenic activities, in different tumors [4]. WW domain-containing transcription regulator 1 (TAZ) is a downstream nuclear effector in the Hippo pathway that induces the proliferation and inhibits the apoptosis of cancer cells [5, 6]. A large number of studies have shown that high expression of TAZ can cause Epithelial-mesenchymal transition (EMT), inhibit apoptosis of cancer cells, and increase the number of cancer stem cells in vitro [7, 8]. Knockdown of TAZ expression can have a negative effect on the migration, invasion, and tumorigenesis of cancer cells in nude mice [9, 10]. Nevertheless, little research has focused on the role of TAZ in the Hippo pathway in CC. In previous studies, TAZ levels were observed to increase in cervical cancer and its microenvironments [11]. However, the precise mechanism of this process in CC cells remains uncertain.

The PD-1/PD-L1 axis immune checkpoint pathway can induce immune evasion of cancer cells and thus inhibit the immune response in various solid tumors, including CC [12]. Overexpression of PD-L1 has been found in human CC [13, 14]. PD-L1 can not only mediate T cell suppression but also promote cancer cell growth and invasion [15]. Early studies have indicated that the Hippo pathway can affect the immune response to cancer and the recruitment and activation of immune cells [16, 17]. The upstream factors mammalian STE20-like kinase 1 and 2 (MST1/2) and large tumor suppressor 1 and 2 (LATS1/2) can inhibit PD-L1 expression, while TAZ and YAP induce high expression of PD-L1 in breast and lung cancer cell lines [18]. Comparison results indicate that TAZ activity can affect PD-L1 expression in cancer cell lines and that the TAZ/YAP/TEAD pathway increases PD-L1 promoter activity [18]. However, no research has demonstrated the correlation of TAZ and PD-L1 in CC and their effects on the tumor characteristics of CC.

Therefore, it is important to evaluate the effects of TAZ and PD-L1 on CC. We showed that TAZ is upregulated and can regulate cell proliferation, invasion, apoptosis, and clinical significance through PD-L1 in the CC in our study. Additionally, significant correlation between TAZ expression and the infiltration levels of immune cells and the expression of PD-L1 were observed in this research.

## Materials and methods

### Cell culture

The cervical cancer cell line HeLa (ACC, USA) was maintained in minimal essential medium (MEM) (HyClone™; Thermo Fisher Scientific, Inc., Waltham, MA, USA) supplemented with 10% fetal bovine serum (FBS) (HyClone™) at 37 °C in a humidified atmosphere of 95% air and 5% CO_2_.

### Cell transfection

At 24 h prior to transfection, HeLa cells were plated into 6-well plates at a density of 5 × 10^5^ per well and were then transfected with 100 pmol of TAZ siRNA or nonspecific sequence control (NC) using Lipofectamine 2000 (Invitrogen, USA) according to the manufacturer’s protocol. PD-L1 siRNA was transfected in a similar manner. The untransfected HeLa cells were also a negative control.

The coding sequence (CDS) of the human TAZ gene was amplified from the cDNA of the cervical cancer cell line SiHa by polymerase chain reaction (PCR) using the following primers:

TAZ-F: CCCAAGCTTATGCCTCTGCACGTGAAG HindIII.

TAZ-R: CCGGAATTCTCTCCCAGGCTGGAGGTG EcoRI.

A pcDNA3 eukaryotic expression vector (Invitrogen, San Diego, CA, USA) was used to establish stably transfect cells overexpressing TAZ. To construct pcDNA3-TAZ, the full-length human TAZ gene pB4 was digested with EcoRI and then inserted into an EcoRI-cleaved pcDNA3 vector. Cells were transfected with pcDNA3 or pcDNA3-TAZ using the lipofection technique according to the manufacturer’s protocol (Gibco BRL, Life Technologies, Rockville, MD, USA).

### Quantitative real-time polymerase chain reaction (RT-qPCR)

Total RNA was extracted using TRIzol^®^ (Transgene, China) from parental and transfected HeLa cells, followed by isopropanol precipitation and chloroform extraction. cDNA was synthesized using the Reverse Transcriptase system (Invitrogen; Thermo Fisher Scientific, Inc.), in accordance with the manufacturer’s protocol. RT-PCR was performed using an iCycler™ Real Time system (Bio-Rad Laboratories, Richmond, CA, USA) using the SYBR Premix EX Tag Master mixture kit (Takara Bio, Inc.) according to the manufacturer's protocol. The quantification of gene transcription was normalized to GAPDH mRNA. The primers used in this study were as follows:

TAZ: Primer F 5′ TCATCACCGTGTCCAATC 3′.

Primer R 5′ CTGAAGAAGTGGGAGTGTAG 3′.

GAPDH: Primer F 5′ CACCCACTCCTCCACCTTTG 3′.

Primer R 5′ CCACCACCCTGTTGCTGTAG 3′.

The samples were amplified in different wells and run in triplicate. The relative expression of genes was compared with the 2^−ΔΔCt^ relative quantification method.

### CCK-8 assay

The effects of TAZ on HeLa cell proliferation were measured by Cell Counting Kit-8 (CCK-8) (Sigma–Aldrich, USA). HeLa cells were plated at a density of 5 × 10^3^ cells per well in 96-well plates 24 h after transfection. Thereafter, 10 µl CCK solution and 100 µl fresh medium were added to each well, and the plates were incubated in the dark at 37 °C for 2 h. After incubation, the absorbance at 452 nm was measured using a Multiskan Go spectrometer (Thermo Fisher, USA).

### Transwell invasion assay

A total of 1 × 10^5^ cells per well in triplicate were plated in the upper chamber of a 24-well plate (pore size 8 µm, Corning, USA) containing 200 µl of serum-free MEM medium. For the invasion assay, the base of the upper chambers was coated with extracellular Matrigel (BD Biosciences, USA) to serve as a chemoattractant. Cells were allowed to invade for 24 h. The Transwell chamber was then removed, the culture solution in the Transwell chamber was discarded, and the chamber was washed twice with calcium-free phosphate-buffered saline (PBS). After that, the chamber was fixed in methanol solution for 30 min and stained with 0.1% crystal violet for 20 min at room temperature. The chamber was washed several times with PBS, and the upper chamber liquid was aspirated. The unmigrated cells in the upper layer were gently wiped off using a cotton swab. The microporous membrane was removed carefully with small tweezers and dried with the bottom side up. Next, the membrane was transferred to a glass slide and sealed with neutral gum. Images were observed and collected by an inverted optical microscope (Keyence, Osaka, Japan).

### Annexin V/propidium iodide apoptosis assay

Apoptosis assays were performed using a commercially available kit, per the manufacturer’s protocol. HeLa cells (control), including the negative control (NC) and cells carrying TAZ siRNA, PD-L1 siRNA, or the TAZ plasmid, or cotransfected with the TAZ plasmid and PD-L1 siRNA were plated at 5 × 10^5^/well in each well of 6-well plates in duplicate. After 24 h, the cells were harvested by trypsinization and suspended in PBS. The cells were subsequently suspended in 500 µl binding buffer (BD Biosciences, USA). Later, 5 µl annexin-V fluorescein isothiocyanate (FITC) (BD Biosciences, USA) and 5 µl propidium iodide (PI) (both purchased from BD Biosciences, USA) were added, and the cells were incubated for 20 min in the dark. Finally, the samples were analyzed using flow cytometric analysis.

### Western blotting

Antibodies against Bcl-2, Caspase-3, PD-L1, MMP-2 and MMP-9 were purchased from Abcam Group, Inc. The information regarding the antibodies is summarized in Table 1. The normal and transfected cells were scraped on ice, collected by centrifugation (12,000×*g*, 10 min, 4 °C) for protein extraction, incubated with freshly prepared RIPA lysis buffer for 15 min, and then quantified with a bicinchoninic acid (BCA) kit (Nanjing KeyGen Biotech. Co., Ltd.). The protein sample was mixed with loading buffer and boiled for 8 min. Subsequently, the sample was separated on a 10% SDS–PAGE gel and electrotransferred onto PVDF membranes (Merck Millipore). The membranes were incubated for 1 h at room temperature with 5% fat-free milk in Tris-buffered saline containing Tween-20, followed by incubation overnight at 4 °C with primary antibodies. Protein bands were captured by a Li-Cor Odyssey Imaging system (LI-COR Biosciences, USA).

**Table 1:** Antibodies

Primary antibodies	Clonality	Catalogue number	Company	Species	Dilution	Diluent
MMP2	Monoclone	66366-1-Ig	Proteintech	Mouse	1:1000	Non-fat milk
MMP9	Monoclone	ab76003	Abcam	Rabbit	1:500	Non-fat milk
Bcl-2	Monoclone	ab32124	Abcam	Rabbit	1:1000	Non-fat milk
Caspase-3	Monoclone	Ab32351	Abcam	Rabbit	1:500	Non-fat milk
PD-L1	Monoclone	Ab205961	Abcam	Rabbit	1:500	Non-fat milk
GAPDH	Monoclone	60004-1-Ig	Proteintech	Mouse	1:500	Non-fat milk

### Oncomine database analysis

The expression levels of the TAZ and PD-L1 genes in cervical cancer were identified in the Oncomine database (https://www.oncomine.org/resource/login.html). The threshold was determined according to the following values: *P* value < 0.05.

### UALCAN

UALCAN (http://ualcan.path.uab.edu/) is a web-based tool that provides in-depth analyses of transcriptome data from The Cancer Genome Atlas (TCGA) and MET500 data. UALCAN was used to investigate the expression of TAZ and PD-L1 and the association between TAZ and PD-L1 and various clinicopathological parameters of lung CC.

### Gene expression profiling interactive analysis (GEPIA) and R2 database

GEPIA (http://gepia.cancer-pku.cn/index.html) is a user-friendly web portal for gene expression analysis based on TCGA and GTEx data. The relationships between hepcidin and PD-1, PD-L1 and CTLA-4 were determined using Spearman’s correlation coefficient in “correlation analysis”. Additionally, to verify the correlation of TAZ and PD-L1 in patients with CC, an R2 database was used to analyze the relationship of TAZ and PD-L1.

### Tumor immune estimation resource (TIMER)

TIMER (https://cistrome.shinyapps.io/timer/), an interactive web portal, performs comprehensive analysis on the infiltration levels of different immune cells. In the present study, TAZ expression in multiple types of cancer was evaluated through the “Diff Exp” module. The correlation of TAZ and immune cell infiltration in CC was analyzed in TIMER. The “Gene” module was used to investigate the relationship between TAZ expression and immune cell infiltration levels (B cells, CD8 + T cells, CD4 + T cells, neutrophils, macrophages, and dendritic cells) using the TCGA database.

### Statistic analysis

All statistical analyses were completed by using SPSS Inc. (Chicago, IL, USA). The Results are presented as the mean ± standard deviation (SD) or standard error of the mean. Statistical significance was determined using ANOVA analysis followed by Tukey's post hoc test. The Spearman rank order correlation was used for the pairwise correlation analyses of expression between proteins. P < 0.05 was considered to indicate a statistically significant value.

## Result

### TAZ and PD-L1 are upregulated in CC

**Fig. 1:**
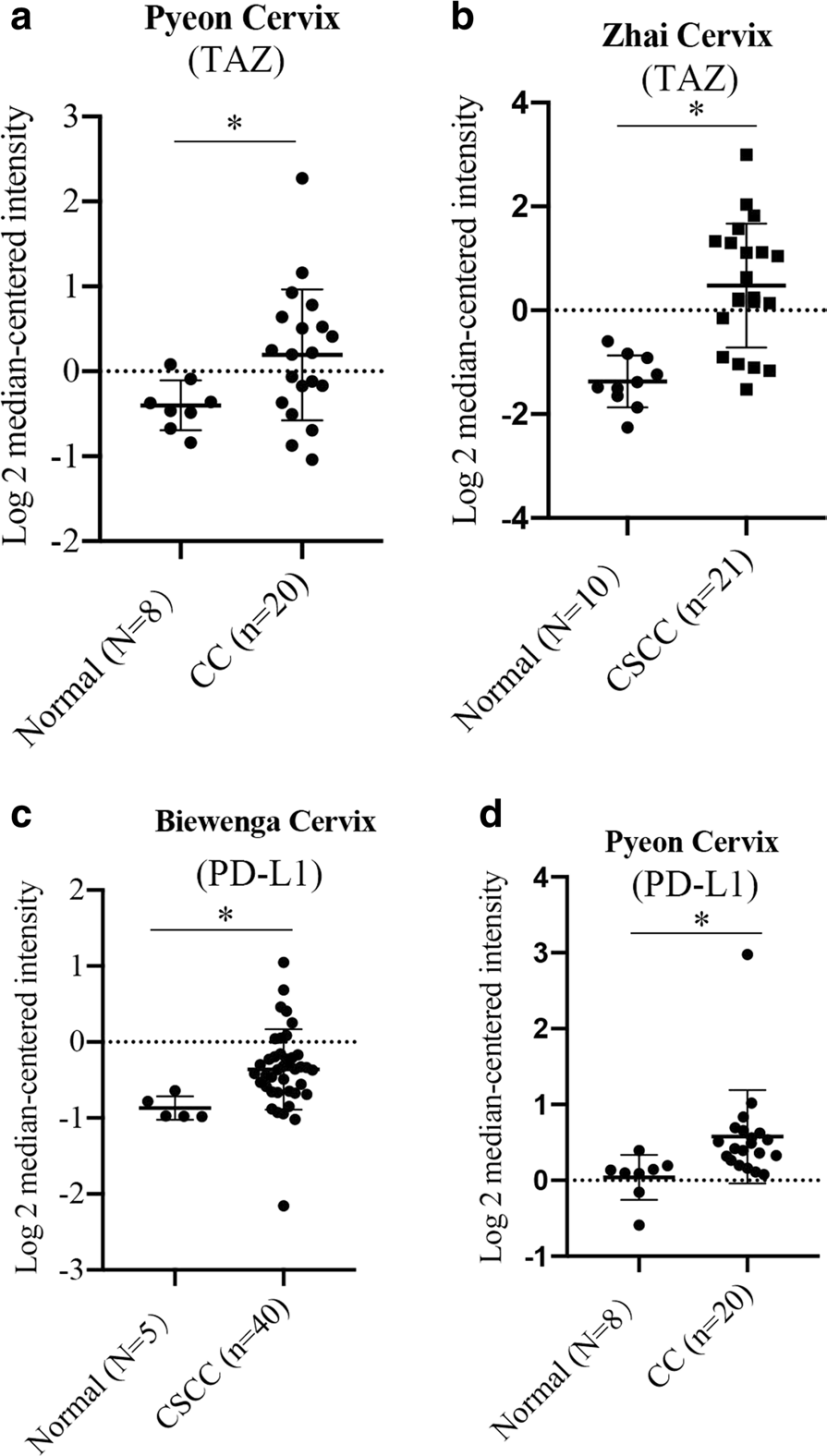
Expression of TAZ and PD-L1 in CC. **A** and **B** The Oncomine database showed that the expression of TAZ was higher in the CC and CSCC groups than in the normal cervix group. **C** and **D** CC and CSCC demonstrated increased expression of PD-L1 compared with normal groups. *P < 0.05 vs. the control group. CC, cervical cancer. CSCC, cervical squamous cell cancer

To observe the difference of expression of TAZ and PD-L1, a comprehensive analysis of hepcidin expression profiles was conducted using publicly assessable datasets from Oncomine database. The expression of TAZ mRNA was found to be increased in the CC group compared with the normal group (Fig. 1A and B). We also found that PD-L1 expression was higher in CC tissues from 2 different cohorts (Fig. 1C and D).

### Expression of TAZ and PD-L1 and clinical parameters of CC patients

**Fig. 2:**
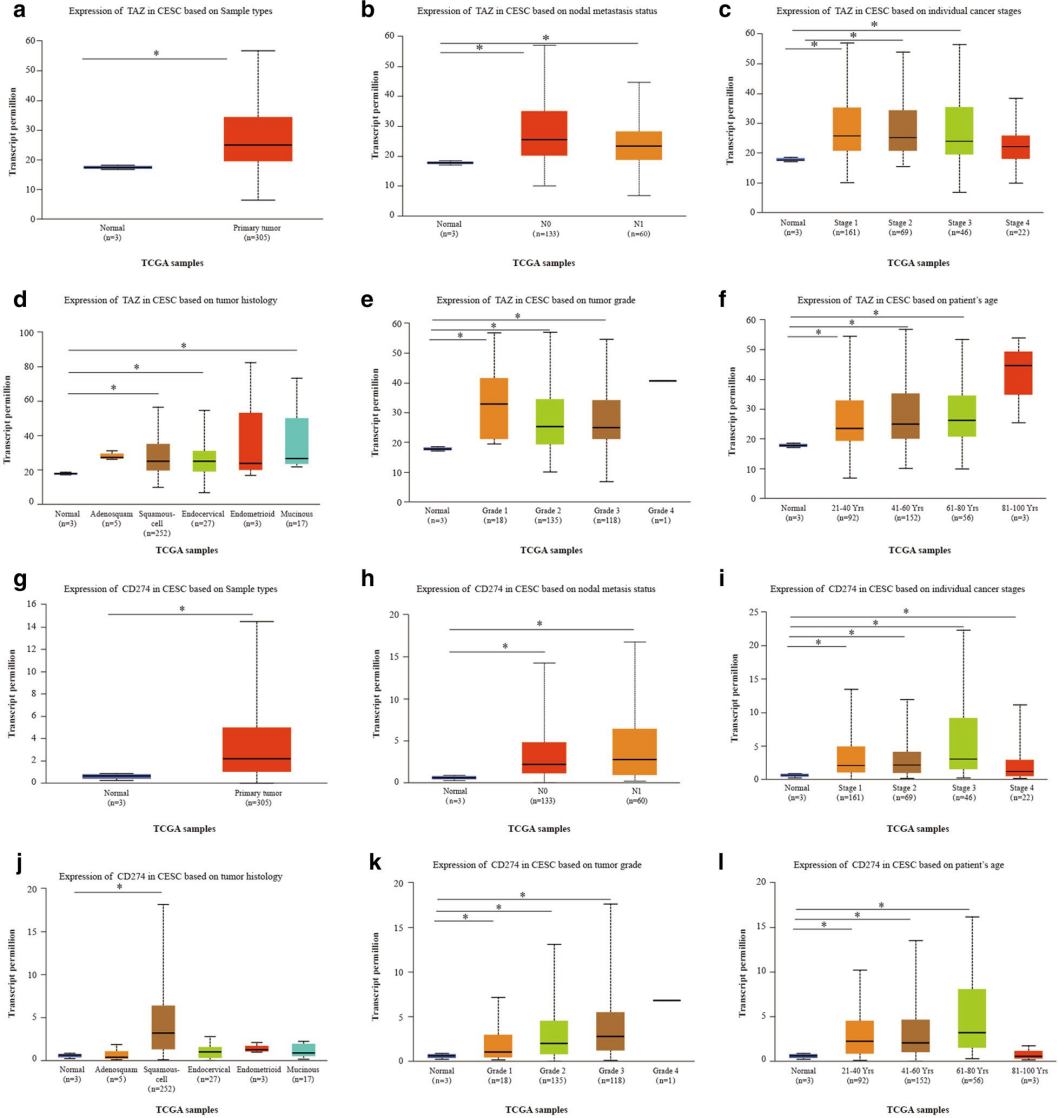
Box plots evaluating TAZ and PD-L1 expression among different groups of patients based on clinical parameters using the UALCAN database. Analysis is shown for sample types (**A** and **G**), metastasis (**B** and **H**), cancer stage (**C** and **I**), tumor histology (**D** and **J**) tumor grade (**E** and **K**) and age (**F** and **I**). *P < 0.05

Considering of the high expression of TAZ and PD-L1, we then focused our investigation on CC and explored the clinical significance of TAZ and PD-L1 expression related to patient survival and disease progression. By using the UALCAN online tool, we then investigated TAZ and PD-L1 expression among groups of patients according to different clinical parameters. The expression of TAZ and PD-L1 was increased in Cervical squamous cell carcinoma and endocervical adenocarcinoma.

(CESCs) compared with normal controls (Fig. 2A and G). According to metastasis status, TAZ and PD-L1 expression were significantly upregulated in CESC samples classified as N0 and N1 compared to the corresponding normal controls; however, the expression of TAZ was lower in patients classified as N1 than in patients classified as No (Fig. 2B and H). Regarding tumor stage, a significant increase in TAZ expression was observed in CESC patients in stages 1, 2, 3, and 4, and a significantly increased expression of PD-L1 was observed in CESE patients in stages 1, 2, and 3 (Fig. 2C and I). Based on tumor histology, TAZ expression was higher in patients with squamous, endocervical, and mucinous cell types (Fig. 2D). PD-L1 expression was higher in the squamous cell type of CESC (Fig. 2J). Upregulation of TAZ and PD-L1 expression was observed in the grade 1, 2, and 3 CESC groups compared to the normal controls (Fig. 2E and K). In terms of age, the TAZ level was significantly elevated in CESC patients from different age groups (21–40 years, 41–60 years, 61–80 years) (Fig. 2F). In addition, PD-L1 expression was dramatically increased in CESC patients aged 21–40 years, 41–60 years, and 61–80 years (Fig. 2L). These results suggest that there is a significant correlation between the expression of TAZ and PD-L1 and the clinical parameters of CESC patients.

### Methylation of TAZ and PD-L1 in CC patients

**Fig. 3:**
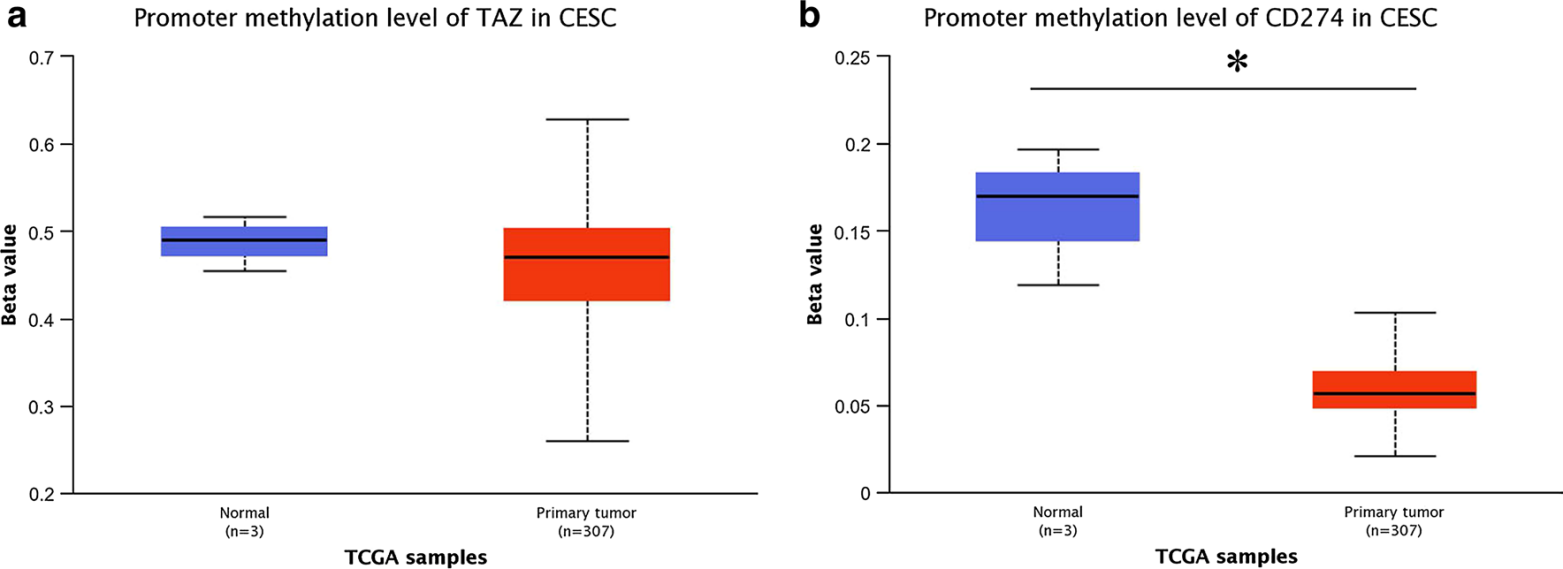
The methylation of TAZ and PD-L1 on CC. **A** The UALCAN database showed that the level of methylation of TAZ was higher in the CC group than in the normal cervix group. **B** The PD-L1 methylation level was higher in the CC group than in the normal group. *P < 0.05 vs. the control group. CC, cervical cancer

We used the UALCAN database to observe the methylation levels of TAZ and PD-L1. The methylation levels of the TAZ promoter tended to be higher in normal controls than in CESC patients (Fig. 3A). Similarly, PD-L1 promoter methylation levels were dramatically higher in the normal controls than in the CESC groups (Fig. 3B). These results indirectly demonstrated that the CESC groups showed higher expression of TAZ and PD-L1.

### Correlation analysis between TAZ expression and infiltrating immune cells

**Fig. 4:**
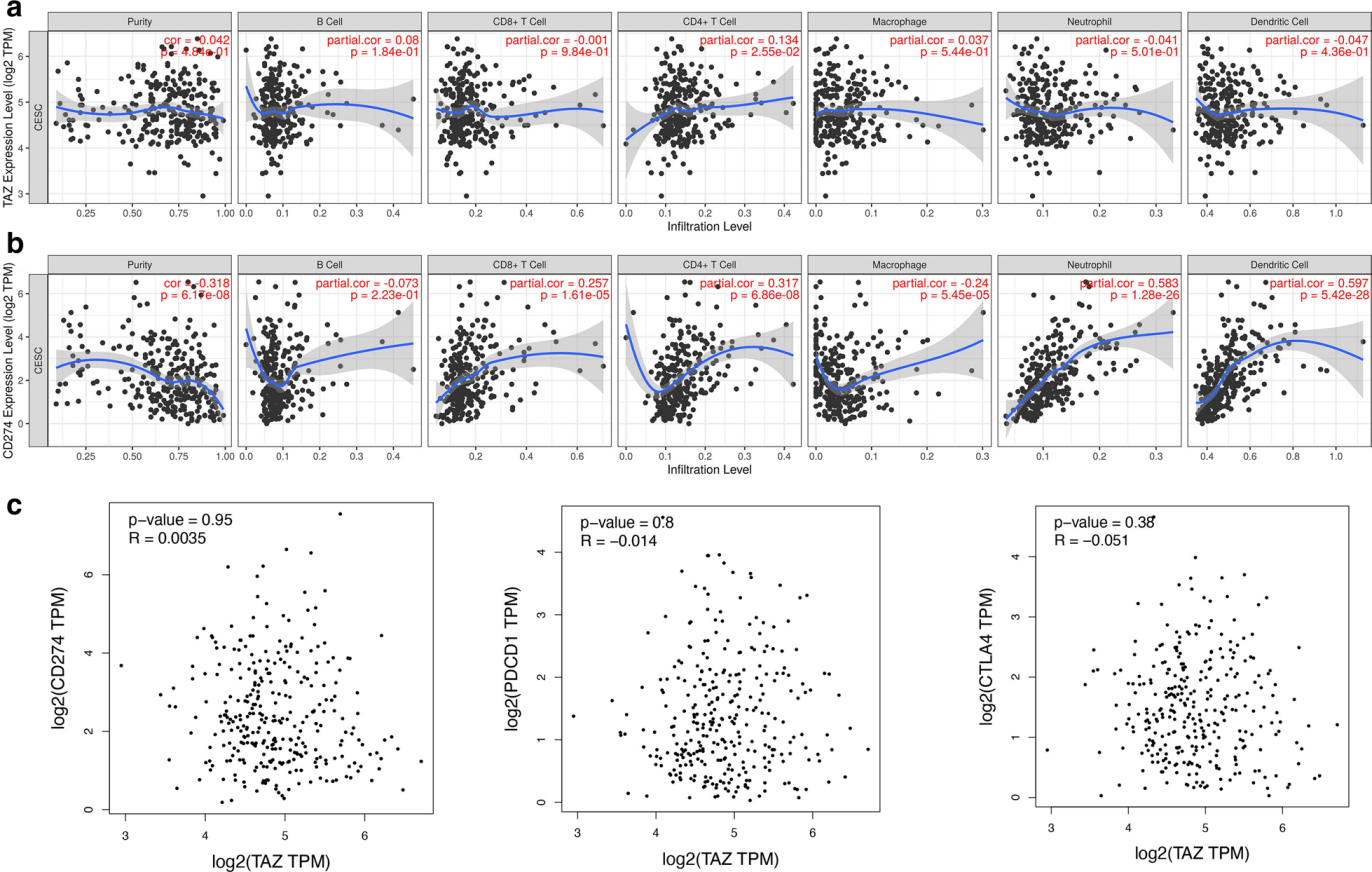
Correlation of TAZ expression with immune infiltration level in CC. **A** TAZ expression is significantly negatively related to tumor purity and has significant positive correlations with infiltrating levels of CD4 + T cells in CC, other than CD8 + T cells, B cells, macrophages, neutrophils, and dendritic cells. **B** PD-L1 expression has significant positive correlations with infiltrating levels of CD4 + T cells in CC, CD8 + T cells, macrophages, neutrophils, and dendritic cells. **C** The GEPIA database indicated a positive correlation of TAZ and PD-L1 (CD274) and a negative association between TAZ and PD-1 (PDCD1) and CTLA4 in CC. *P < 0.05. CC, cervical cancer

To further assess the effect of TAZ and PD-L1 on the tumor microenvironment (TME), we estimated the correlation between TAZ and PD-L1 and immune cells by using the TIMER database. We analyzed the correlation between TAZ expression and B cells, CD4 + T cells, CD8 + T cells, neutrophils, macrophages, and dendritic cells. The results showed that there was a significant positive relationship between TAZ expression levels and the infiltration of CD4 + T cells, and there were no significant correlations between the levels of TAZ and B cells, CD8 + T cells, macrophages, neutrophils, and dendritic cells in CESCs (Fig. 4A). We also analyzed the relationship between PD-L1 expression and B cells, CD4 + T cells, CD8 + T cells, neutrophils, macrophages, and dendritic cells. The result demonstrated that the expression of PD-L1 was associated with CD4 + T, cells CD8 + T cells, macrophages, neutrophils, and dendritic cells in CESCs, but was not correlated with B cells (Fig. 4B).

### Correlation analysis between TAZ expression and immune checkpoints

**Fig. 5:**
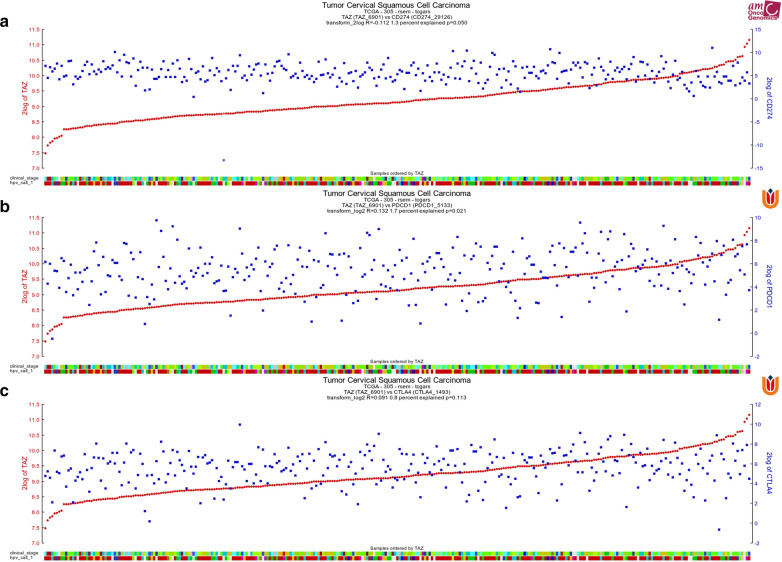
Correlation of TAZ expression with immune checkpoints. **A** The R2 database showed a positive association between TAZ and PD-L1 in CC. **B** The R2 database showed a positive association between TAZ and PD-1 in CC. **C** The R2 database showed a non-significant positive association between TAZ and PD-L1 in CC

Based on high expression of TAZ and PD-L1, the significant association between CD4 + T cells and the expression of TAZ and PD-L1 in CC, we also want to know whether there is a significant correlation between TAZ and PD-L1. We found that TAZ expression was positively correlated with PD-L1 and PD-1 expression by utilizing the R2 database. However, there is no significant correlation between TAZ and CTLA4 expression via R2 database (Fig. 5A–C). Additionally, we also used the GEPIA database to investigate the correlation between TAZ expression and immune checkpoints (PD-L1, PD-1, and CTLA4). We found that TAZ expression was positively associated with PD-L1; however, it was negatively correlated with PD-1 and CTLA4 (Fig. 4C).

### PD-L1 is upregulated by TAZ in CC cells

**Fig. 6:**
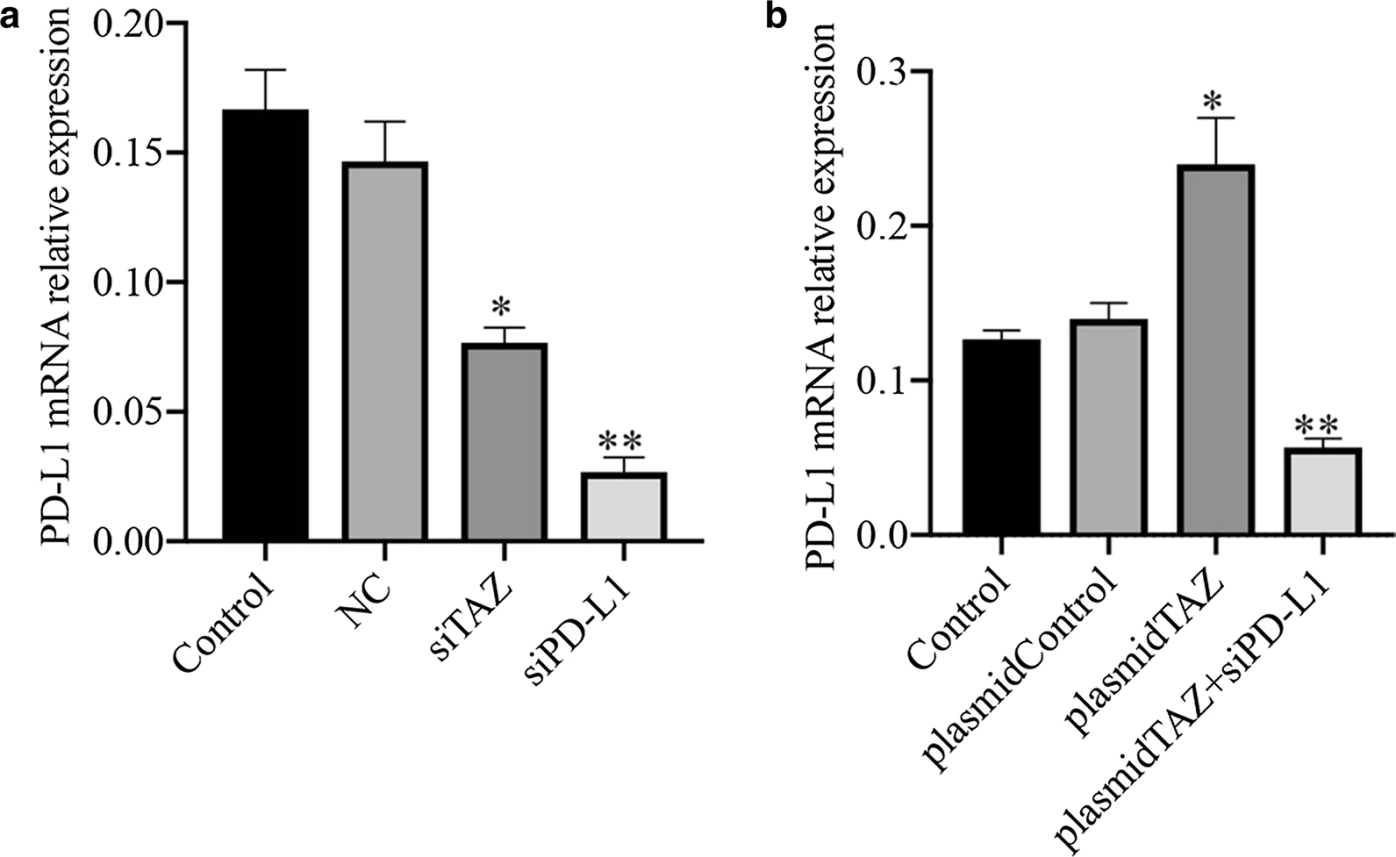
The expression of PD-L1 mRNA in CC. **A** The level of PD-L1 mRNA was lower in the TAZ siRNA and PD-L1 siRNA groups than in the control groups. **B** PD-L1 expression was upregulated in the TAZ plasmid groups compared with the control groups, and PD-L1 expression was decreased in the TAZ plasmid and PD-L1 siRNA groups compared with the TAZ plasmid groups. *P < 0.05. CC, cervical cancer

**Fig. 7:**
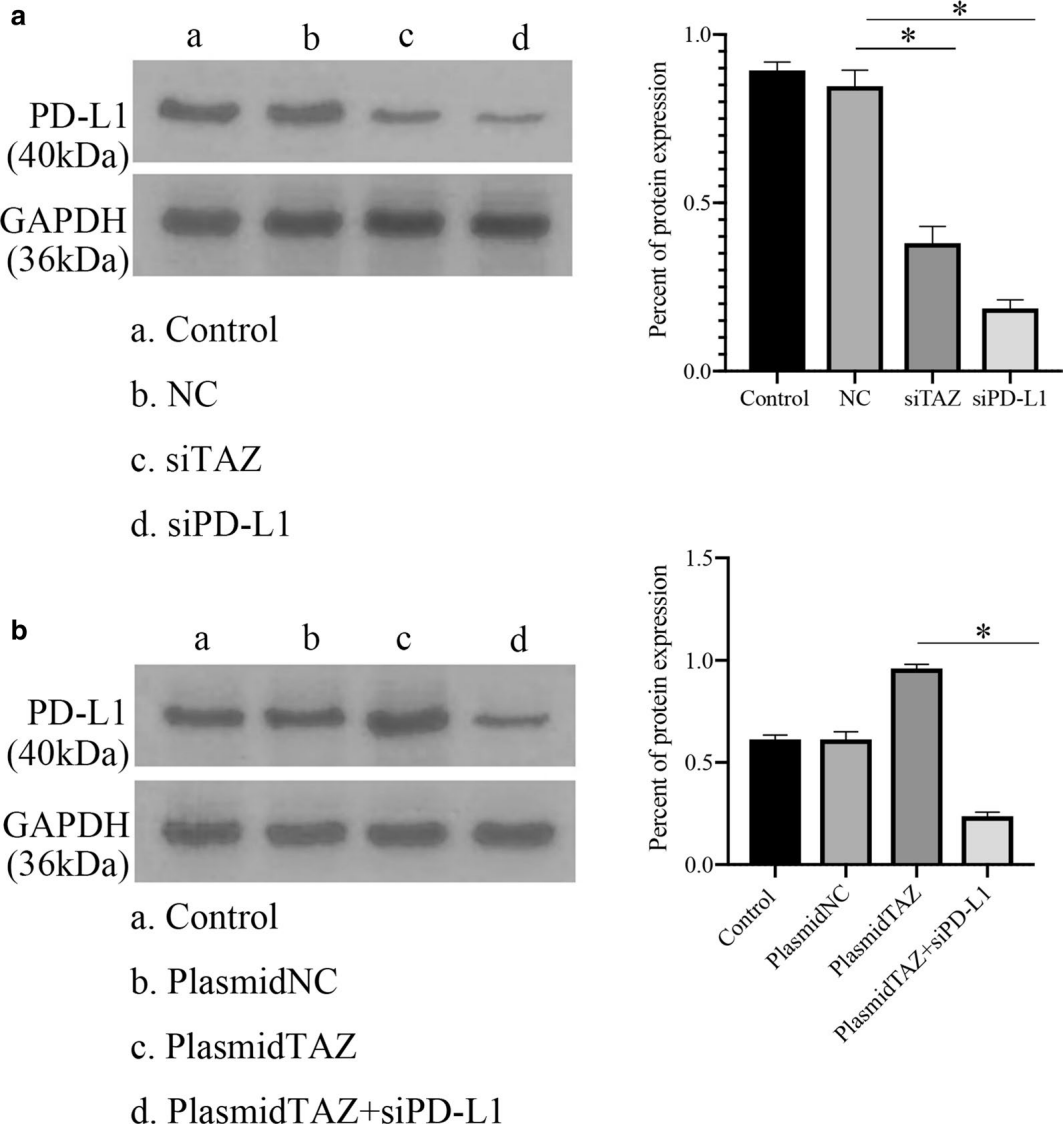
The protein expression of PD-L1 in CC. **A** The level of PD-L1 was lower in the TAZ siRNA and PD-L1 siRNA groups than in the control groups. **B** PD-L1 expression was upregulated in the TAZ plasmid groups compared with the control groups, and PD-L1 expression was decreased in the TAZ plasmid and PD-L1 siRNA groups compared with the TAZ plasmid groups. *P < 0.05. CC, cervical cancer

Given the positive correlation between TAZ and PD-L1, we want to know whether PD-L1 can be regulated by TAZ. We transfected TAZ siRNA into HeLa cells. PD-L1 was significantly decreased by TAZ siRNA at the mRNA level, as shown by qRT–PCR, in contrast with the normal and negative control groups (Fig. 6A). When we transfected TAZ plasmid into HeLa cells, the level of PD-L1 mRNA was upregulated compared with the normal and negative control groups (Fig. 6B). Furthermore, when we transfected TAZ plasmid and PD-L1 siRNA into CC cells, the mRNA level of PD-L1 was downregulated compared with that in the TAZ plasmid groups (Fig. 6B). Similarly, the protein expression of PD-L1 was inhibited in groups of TAZ siRNA and PD-L1 siRNA compared with normal control groups (Fig. 7A). In addition, the expression of PD-L1 was downregulated in groups of TAZ plasmid and PD-L1 siRNA compared with that in the TAZ plasmid groups (Fig. 7B).

### TAZ improves cell proliferation of CC Cells via upregulating PD-L1

Previous studies have shown that TAZ can make an important role on CC, and we also found there is a positive correlation between TAZ and PD-L1. Therefore, we next evaluated whether TAZ can regulate the characteristics of cancer cells via PD-L1.

**Fig. 8:**
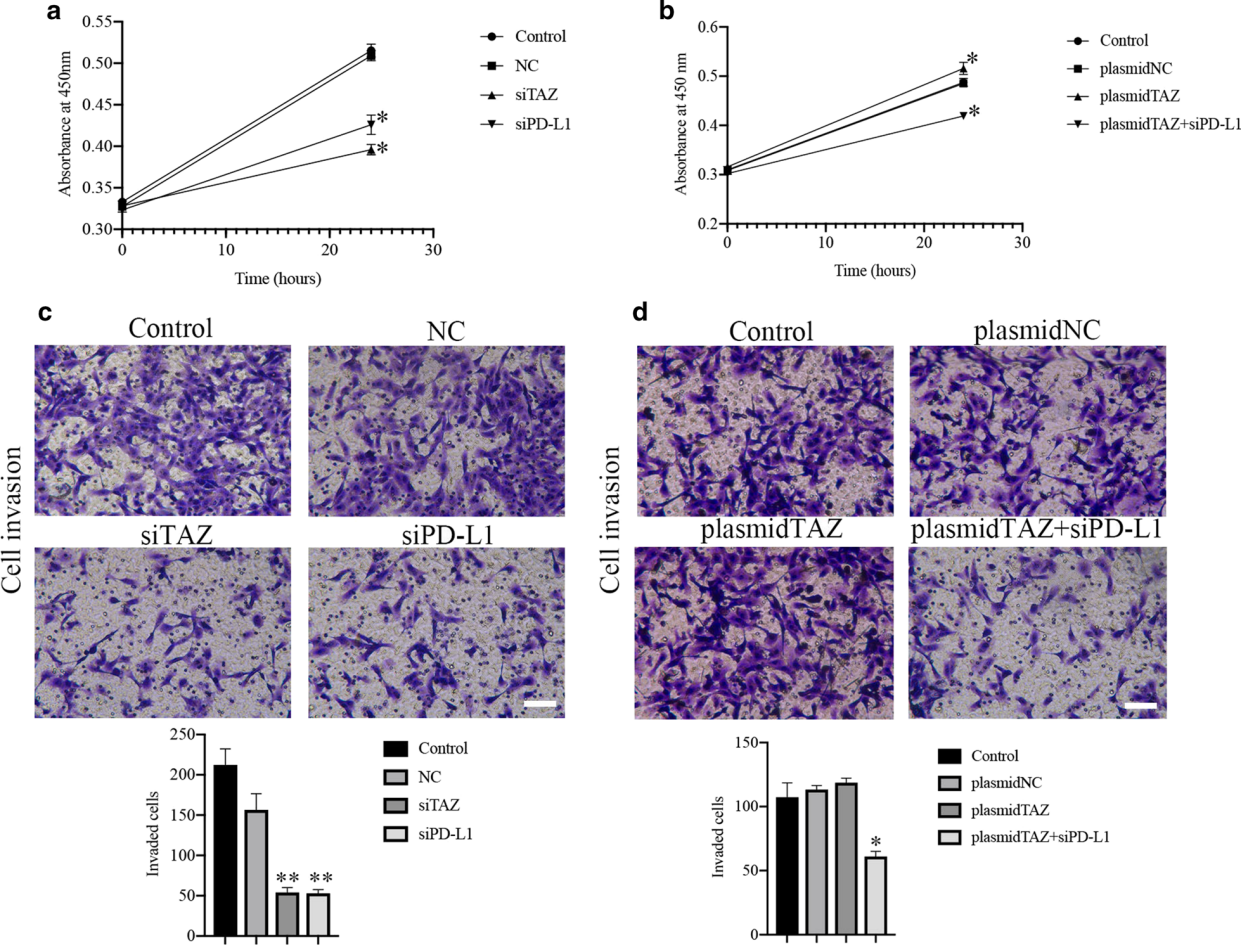
TAZ and PD-L1 regulates CC progression. **A** Representative images and quantification of CCK-8 assays indicating the growth and invasion of HeLa cells stably transfected with empty vector (mock), TAZ siRNA, and PD-L1 siRNA. **B** Representative images and quantification of CCK-8 assays indicating the growth and invasion of HeLa cells stably transfected with empty vector (mock), TAZ plasmid, and PD-L1 siRNA and TAZ plasmid. **C** and **D** Representative images of Matrigel invasion assays showing the growth and invasion of HeLa cells stably transfected with TAZ siRNA, PD-L1 siRNA, TAZ plasmid, and PD-L1 siRNA and TAZ plasmid. *P < 0.05

To determine the influence of TAZ on CC cells, we used a CCK-8 assay to assess cell proliferative capacity. There was a decrease in cell proliferation in the TAZ siRNA and PD-L1 siRNA groups compared with the control and negative control groups (Fig. 8A, P < 0.05). The cell groups transfected with the TAZ plasmid showed higher cell growth ability than the control and negative groups (Fig. 8B, P < 0.05). The cell proliferation capacity in the TAZ plasmid and PD-L1 siRNA cell groups was decreased compared with that in the TAZ plasmid group (Fig. 8B, P < 0.05).

### TAZ improves cell invasion of CC cells via upregulating PD-L1

**Fig. 9:**
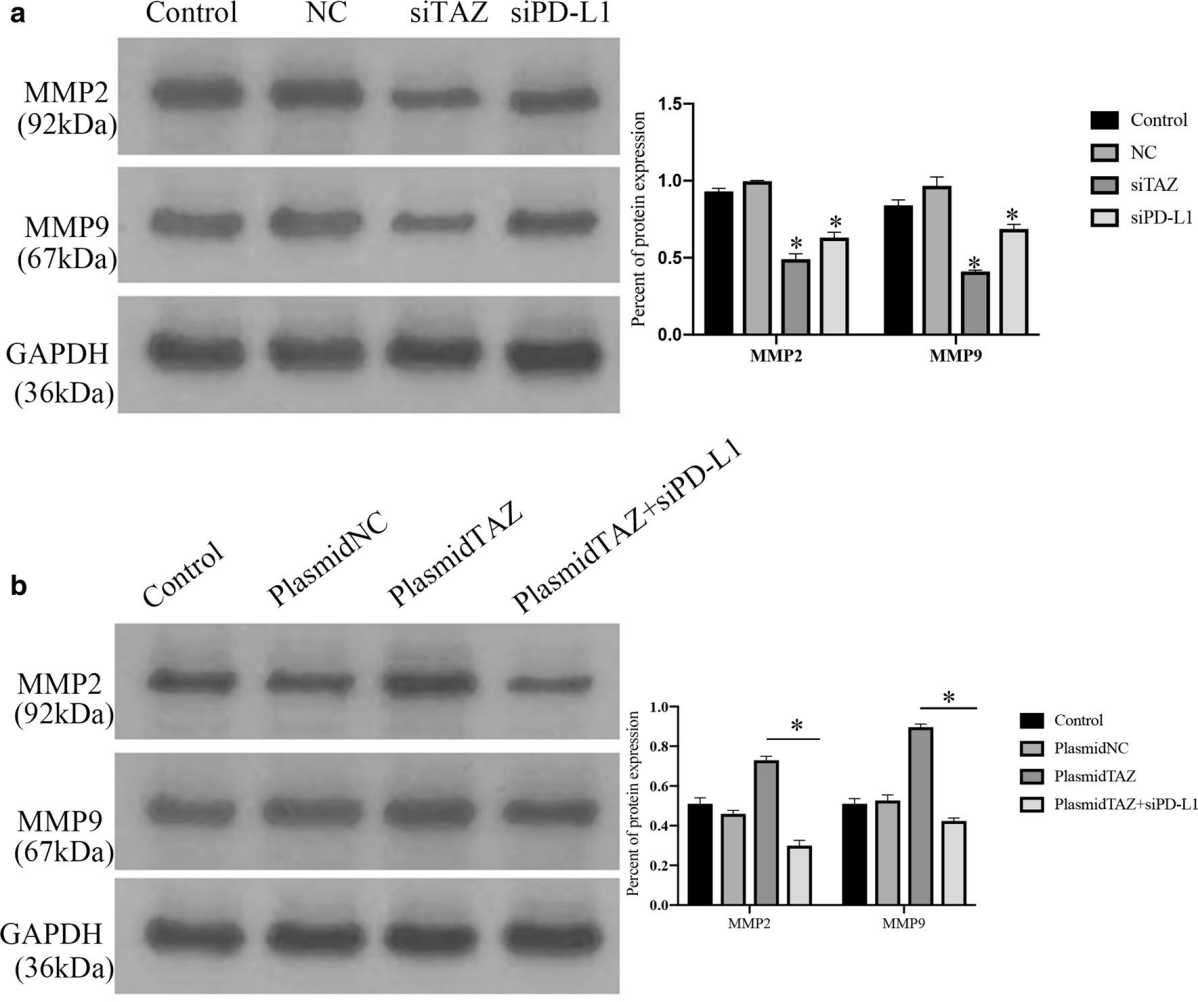
TAZ promotes metastasis of CC cells associated with PD-L1. **A** and **B** The expression of MMP-2 and MMP-9 was measured by western blotting assay

The invasion ability of the cell groups transfected with TAZ siRNA and PD-L1 siRNA was weakened compared with that of the control and negative groups (Fig. 8C, P < 0.05) according to the Transwell assay. There was an increase in cell invasion ability in the TAZ plasmid groups, even if the statistical analysis was not significant (Fig. 8D). In addition, the groups transfected with the TAZ plasmid and PD-L1 siRNA showed lower invasion capacity than the groups transfected with only the TAZ plasmid (Fig. 8D, P < 0.05). Additionally, the protein expression of MMP-2 and MMP-9 in was decreased in groups of TAZ siRNA and PD-L1 siRNA compared with that of the control and negative groups (Fig. 9A, P < 0.05). the groups transfected with the TAZ plasmid and PD-L1 siRNA showed lower expression of PD-L1 than the groups transfected with only the TAZ plasmid (Fig. 9B, P < 0.05).

### TAZ inhibits cell apoptosis of CC cells via upregulating PD-L1

**Fig. 10:**
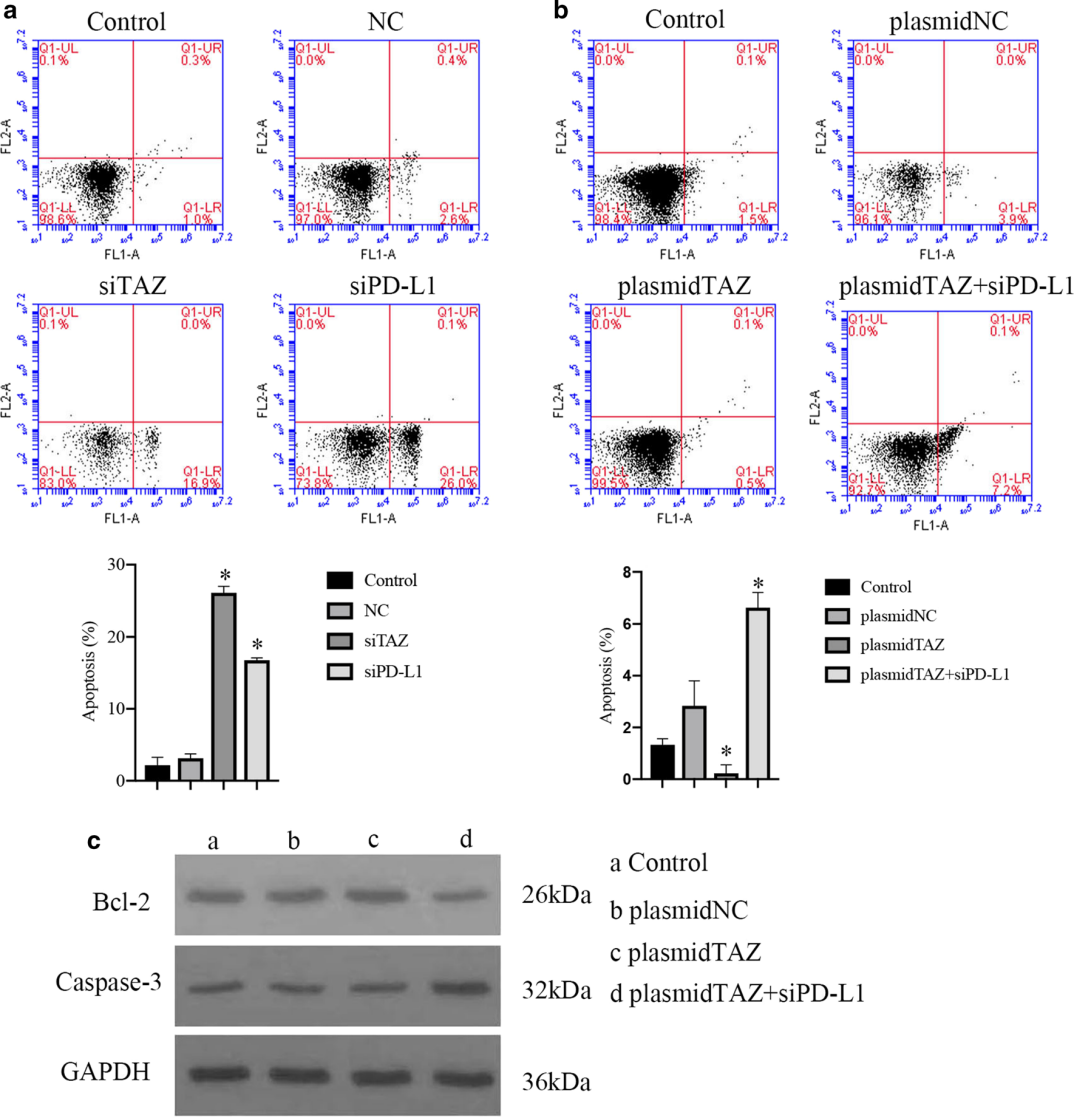
TAZ suppresses apoptosis of CC cells associated with PD-L1. **A** Apoptosis was induced in TAZ siRNA cell group and PD-L1 siRNA cell groups Compared with control cell group. **B** Apoptosis was inhibited in plasmidTAZ cell group Compared with control cell group. Apoptosis was induced in plasmidTAZ and siPD-L1 groups Compared with plasmid TAZ cell group, P < 0.05. **C** The expression of Bcl-2 and Caspase-3 was measured by western blotting assay

The present study further assessed whether TAZ could inhibit apoptosis in CC by regulating PD-L1. An annexin V/PI apoptosis assay was performed using flow cytometry. The percentages of apoptosis in the groups transfected with TAZ siRNA and PD-L1 siRNA were 16.9% and 26.1%, respectively, which were higher than those in the control groups (Fig. 10A, P < 0.05). The percent of apoptosis in the groups transfected with the TAZ plasmid was 0.6%, which was lower than that in the control groups (Fig. 10B, P < 0.05). Additionally, when we transfected TAZ plasmid and PD-L1 siRNA into the cell groups, the percent of apoptosis was increased compared with that in the TAZ plasmid groups (Fig. 10B, P < 0.05).

The study also evaluated the expression of Bcl-2 and Caspase-3, and the expression of Bcl-2 in groups transfected with TAZ plasmid and PD-L1 siRNA was decreased compared with that in the TAZ plasmid groups (Fig. 10C). In contrast, the expression of Caspase-3 was increased in the TAZ plasmid and PD-L1 siRNA groups compared with that in the TAZ plasmid group (Fig. 10C).

## Discussion

CC is considered a common gynecological cancer in women worldwide, with 570,000 reported incident cases documented each year [19]. Therefore, it is of utmost importance to identify novel treatment strategies for CC.

Here, we have defined TAZ, a Hippo signaling transducer and a novel oncogene, as being responsible for CC tumorigenesis. Our data revealed that TAZ is significantly overexpressed in CC and is closely correlated with PD-L1. Notably, TAZ promotes proliferation, anti‐apoptosis, migration, and invasion by regulating PD-L1 in CC. We indicated a novel mechanism of TAZ in CC, which is extremely important for the treatment of CC.

Cancer research in the past decade has indicated that the Hippo pathway regulates cell proliferation, tissue homeostasis, and organ size and is a promoter of tumorigenesis and tumor migration [20, 21]. As a paralog of YAP, TAZ shares 46% homology with YAP. In numerous studies, both YAP and TAZ are referred to as a pair, as many antibodies detect both proteins due to their high similarity. Importantly, YAP and TAZ cannot compensate for each other [22]. Previous studies found that active TAZ increased migration and colony forming ability but had no effect on proliferation. This result was associated with increased expression of E-cadherin, FN1, vimentin, and B-catenin [23]. TAZ can function independently of YAP to enhance DNA synthesis and appears to be important in cell cycle regulation [24]. A previous study analyzed the function of the Hippo pathway in 308 CC patients from The Cancer Genome Atlas (TCGA) and showed that TAZ was continually amplified in CC, and the high genetic expression of TAZ was associated with poor prognosis [12]. In recent years, several tumors, including thyroid carcinoma [25], rectal cancer [26], and colon cancer [27], have shown high levels of TAZ, suggesting that it may function as a promoter in tumorigenesis. Similarly, we also found that TAZ is overexpressed in CC and can induce growth and metastasis and inhibit apoptosis of CC cells.

How does TAZ affect tumorigenesis and tumor migration? A recent study found that the overexpression of several genes in the JAK–STAT3 pathway is induced by active YAP1 and TAZ interacting with the transcription factor TEAD [28]. This pathway controls the response to inflammatory cytokines, is present at high levels in pancreatic tumors, and can lead to PanIN progression in *KRas*^*G12D*^ mice [29, 30]. In addition, TAZ is necessary for TGF-β response elements and maintains the nuclear accumulation of the Smad2/3-Smad4 complex and stem cell self-renewal mediated by TGF-β [31]. Noticeably, YAP and TAZ can have a positive effect on the expression of the immune checkpoint molecule PD-L1, thereby suppressing the antitumor ability of T cells in several different models [32]. Feng et al. demonstrated that the pH of the extracellular environment can affect the activity of Hippo signaling in human lung adenocarcinoma, which induces TAZ to upregulate the level of PD-L1 [33]. PD-L1 or B7-H1 is the major ligand for PD-1 [34]. PD-L1 is expressed in immune cells, including activated T cells, B cells, dendritic cells, macrophages, and various tumor cells [35, 36]. Normally, PD-L1 expression maintains the homeostasis of the immune response. PD-L1 expressed by cancer cells and infiltrating immune cells can bind to PD-1 on T cells and then suppress the functions of T cells in the tumor microenvironment [37, 38]. Prognosis determined by oncological histology is closely related to the expression of PD-L1; therefore, intervention in PD-L1 expression can be the key to tumor therapy [39–42]. Multiple studies identified PD-L1 as a direct transcriptional target of YAP/TAZ/TEAD and showed that YAP and/or TAZ activation upregulates PD-L1 in human breast cancer, NSCLC, mesothelioma and melanoma cells [43]. We characterized the molecular mechanisms by which TAZ enhances PD-L1 expression by binding to the PD-L1 promoter through the TEAD family of transcription factors.

Recent data suggested that PD-L1 is overexpressed in CC cells and can promote the growth and metastasis of CC [44]. PD-L1 is an important biomarker for evaluating CC prognosis and clinical pathological characteristics [45]. Consistent with this, our results also showed that TAZ expression is significantly correlated with PD-L1 expression in CC, and TAZ inhibited the percent apoptosis of cervical cancer cells by regulating PD-L1 and promoted proliferation and metastasis.

Research developments have led to an entirely new class of drugs, antibodies directed against PD-L1/PD-1, which promote the body's immune system to fight cancer. The expression and roles of TAZ and PD-L1 in the progression of CC provide great potential for observing new targeted cancer therapies.

## Data Availability

All data generated or analysed during this study are included in this published article.
